# *Bacillus amyloliquefaciens* RWL-1 as a New Potential Strain for Augmenting Biochemical and Nutritional Composition of Fermented Soybean

**DOI:** 10.3390/molecules25102346

**Published:** 2020-05-18

**Authors:** Raheem Shahzad, Adeeb Shehzad, Saqib Bilal, In-Jung Lee

**Affiliations:** 1 Basic and Applied Scientific Research Center, Imam Abdulrahman Bin Faisal University, P.O. Box 1982, Dammam 31441, Saudi Arabia; rmshahzad@iau.edu.sa; 2 Department of Biology, College of Science, Imam Abdulrahman Bin Faisal University, P.O. Box 1982, Dammam 31441, Saudi Arabia; 3 Department of Clinical Pharmacy, Institute for Research & Medical Consultations (IRMC), Imam Abdulrahman Bin Faisal University, P.O. Box 1982, Dammam 31441, Saudi Arabia; asmsiar@iau.edu.sa; 4 Natural and Medical Sciences Research Center, University of Nizwa, Nizwa 616, Oman; saqib043@yahoo.com; 5 School of Applied Biosciences, Kyungpook National University, Daegu 41566, Korea

**Keywords:** fermented soybean, fermentation, antioxidant activity, amino acid, isoflavones, nutritional composition

## Abstract

Soybean (Glycine max L.) is a good source of natural antioxidants and commonly consumed as fermented products such as cheonggukjang, miso, tempeh, and sufu in Asian countries. The aim of the current study was to examine the influence of novel endophytic bacterial strain, *Bacillus amyloliquefaciens* RWL-1 as a starter for soybean fermentation. During fermentation, the cooked soybeans were inoculated with different concentrations (1%, 3%, and 5%) of *B. amyloliquefaciens* RWL-1. The changes in 2,2-diphenyl-1-picrylhydrazyl (DPPH), 2,2′-azino-bis (3-ethylbenzthiazoline-6-sulfonic acid) (ABTS) radical scavenging activities, total phenolic contents, isoflavones (Daidzin, Genistin, Glycitin, Daidzein, Glycitein, and Genistein), amino acids (aspartic acid, threonine, serine, glutamic acid, glycine, alanine, cysteine, valine, methionine, isoleucine, leucine, tyrosine, phenylalanine, lysine, histidine, arginine, and proline) composition, and minerals (calcium, copper, iron, potassium, magnesium, manganese, sodium, nickel, lead, arsenic, and zinc) were investigated. The level of antioxidants, total phenolic contents, isoflavones, and total amino acids were higher in fermented soybean inoculated with 1% *B. amyloliquefaciens* RWL-1 after 60 h of fermentation as compared to control, 3% and 5% *B. amyloliquefaciens* RWL-1. Additionally, fermented soybean inoculated with 5% *B. amyloliquefaciens* RWL-1 showed the highest values for mineral contents. Changes in antioxidant activities and bioactive compounds depended on the concentration of the strain used for fermentation. From these results, we conclude that fermented soybean has strong antioxidant activity, probably due to its increased total phenolic contents and aglycone isoflavone that resulted from fermentation. Such natural antioxidants could be used in drug and food industries and can be considered to alleviate oxidative stress.

## 1. Introduction

Soybean (Glycine max L.) is the most recognized source of plant protein, which plays a key role for human health. Soybean is a rich source of important nutritive constituents, such as carbohydrates, vitamins, minerals, saponins, isoflavones, flavonoids, and peptides that are of high therapeutic importance [1]. Twelve different kind isoflavones are found in soybeans and soy-containing products. These isoflavones refer to phytoestrogens which play an important role in the inhibition of osteoporosis and cardiovascular diseases due to their estrogenic activities [2]. Several studies have reported that a high soybean intake is associated with a low incidence of breast cancer in Asian countries [3]. At the same time, scientists are attracted to soybean because of its supply for different types of valuable minerals such as calcium, phosphorus, and iron [4]

Soybean is used as some unfermented products such as roasted and fried soybeans, and fermented as soy sauce, tempeh, Chinese douche, sufu, miso, natto, kanjang, doenjang, and cheonggukjang (CGJ) [5]. Fermentation has been used to preserve the spoilable foods since ancient times and the idea behind the process was especially where there was a lack of such foods. Recently, fermentation is applied to enhance active components responsible for improving health [6]. Complex organic compounds are broken down into smaller molecules during fermentation, which exerts physiological functions beyond their dietary properties. Fermentation can also result in a reduction of anti-nutritional components such as oxalic acids, proteinase-inhibitors, urease, and phytic acid [7]. Recent advancements in probiotic formulations revealed that many *Bacillus* strains have probiotic properties. Ushakova et al. [8] directed the importance of biofilms produced by *Bacillus* strains and their promising application in medical, veterinary, and pharmaceutical probiotic formulations. Equally, soybean has been implemented in bioprocessing concepts and yeast bioconversion to generate carotenoids and microbial oil that was further employed for novel oleogels formulation, thereby broadening the range of food applications [9,10]. The formulation technologies of selected potent strains would provide a better understanding of digestion and immune enhancement due to probiotic dietary supplements [11].

Fermented soybean with various microorganisms improves the nutritious properties due to the rise in free isoflavones, resulting in higher concentrations of aglycones, improved minerals, and amino acids [12,13,14,15]. Depending on their chemical forms metabolic fate of soybean isoflavones differ due to the structure itself. The chemical forms of the isoflavones are a limiting factor for absorption in the rumen and intestinal wall, and their metabolites such as aglycones influence the extent of absorption with more readily absorbed and more actively available than highly polar conjugated species [14]. The acetyl and malonyl glycosides are metabolized to glycosides during ingestion, which is hydrolyzed by microbes in the large intestine, to produce their respective aglycones daidzein, genistein, and glycitein [15]. However, an efficient method for safety and functionality enhancement of fermented soy foods is the use of *Bacillus* strains with desirable traits as a starter. Desirable characters of microbes to be used as starter for food fermentation consist of their potential to inhibit the growth of pathogenic microbes, the ability to produce bioactive metabolites and the capability to confer ideal organoleptic properties on fermented foods [16].

Therefore, the current study was carried out with our previously isolated *B. amyloliquefaciens* RWL-1 which was initially isolated from rice seeds and has been reported for antifungal potential and various secondary metabolites (phytohormones and organic acids) and novel compound ((*S*)-2-hydroxy-*N*-((*S*)-1-((*S*)-8-hydroxy-1-oxoisochroman-3-yl)-3-methylbutyl)-2-((*S*)-5-oxo-2,5-dihydrofuran-2-yl)acetamide) production [17,18,19,20]. The objectives of the current study were to systematically evaluate the total phenolic contents and phytochemical substances to get the overall in vitro antioxidant capacities of fermented soybean using *B. amyloliquefaciens* RWL-1. Especially, this study focused on isoflavone contents, total amino acids, and minerals to find the relationships between the production and distribution of bioactive compounds in fermented soybean. In addition, we identified and quantified the correlation of antioxidants and their activities in fermented soybean. Developments from this study should expect to help consumers with suggestions to choose commercially fermented soybean and provide appropriate evidence to use fermented soybean in pharmaceutical, food, and cosmetic industries.

## 2. Results

### 2.1. Total Phenolic Contents

**Figure 1:**
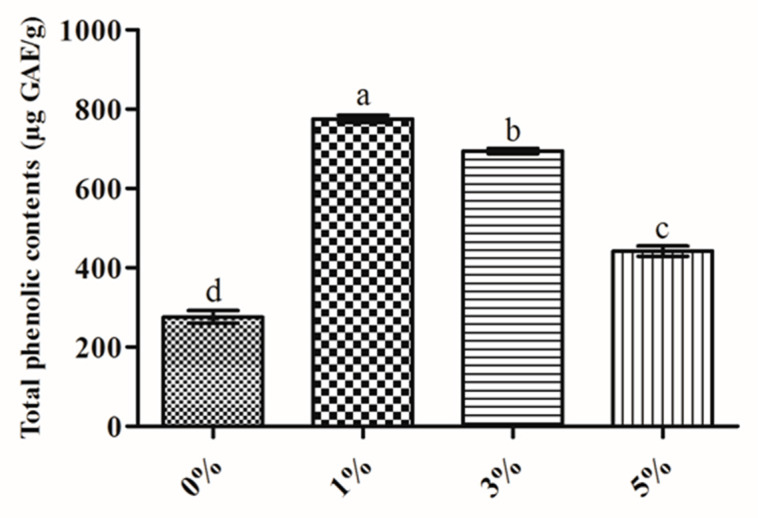
Total phenolic contents of non-fermented and fermented soybean using different concentrations (1%, 3%, and 5%) of the *B. amyloliquefaciens* RWL-1. Values are means of three independents experiments. The letters a, b, c, and d indicate significant differences (*P* ≤ 0.05) by Duncan’s multiple range tests (DMRT).

In the current study, a significant variation (*P* < 0.05) in total phenolic contents was observed among the unfermented and fermented soybean with various percentages of *B. amyloliquefaciens* RWL-1 as summarized in Figure 1. The results revealed that the soybean fermentation with 1% *B. amyloliquefaciens* RWL-1 exhibit a significantly higher amount of total phenolic contents (TPC) with 181.50% increase in soybean fermented with 1% *B. amyloliquefaciens* RWL-1 followed by 151.93% and 60.31% increase in soybean fermented with 3% and 5% *B. amyloliquefaciens* respectively, as compared to control (Figure 1).

### 2.2. Antioxidants

**Figure 2:**
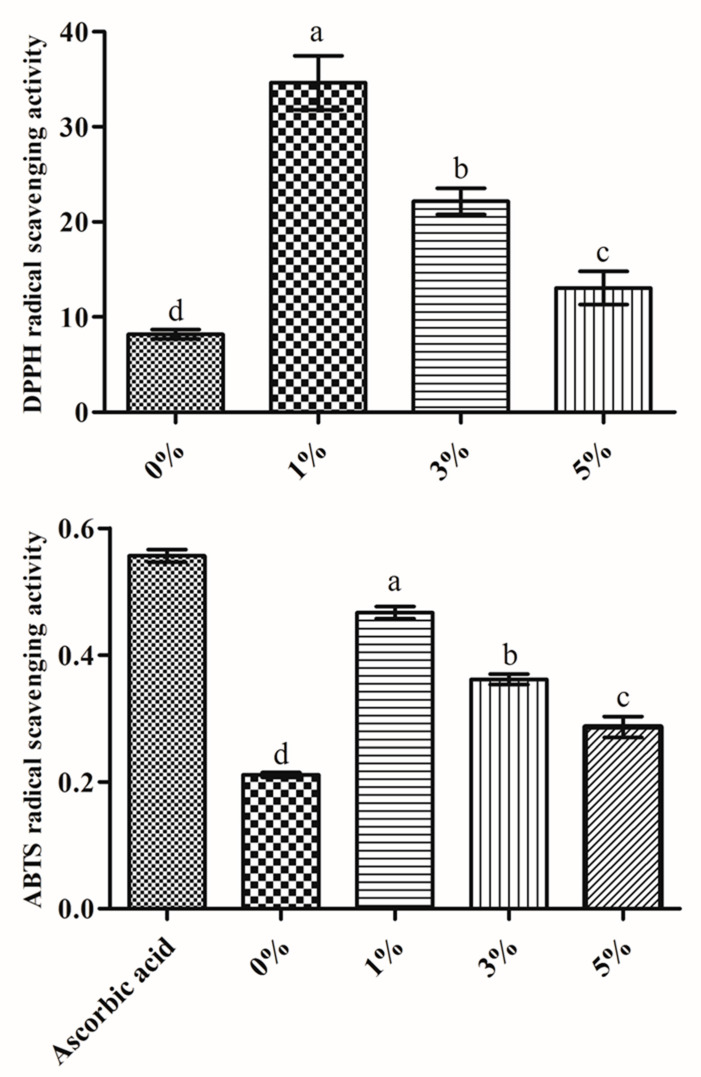
2,2-diphenyl-1-picrylhydrazyl (DPPH) radical-scavenging and 2,2′-azino-bis (3-ethylbenzthiazoline-6-sulfonic acid) (ABTS) radical scavenging activity of non-fermented and fermented soybean using different concentrations (1%, 3%, and 5%) of the *B. amyloliquefaciens* RWL-1. The letters a, b, c, and d indicate significant differences (*P* ≤ 0.05) by DMRT.

In the current study, the *B. amyloliquefaciens* RWL-1 fermentation significantly maximizes the free radical scavenging potential as shown in Figure 2. The results showed that the higher free 2,2-diphenyl-1-picrylhydrazyl (DPPH) scavenging activity was recorded in soybean fermented with 323.39% increase in soybean fermented with 1% *B. amyloliquefaciens* RWL-1 followed by 171.02% and 59.65% increase in soybean fermented with 3% and 5% respectively, as compared to control (Figure 2).

In our study, the *B. amyloliquefaciens* RWL-1 fermentation interestingly enhanced the 2,2′-azino-bis (3-ethylbenzthiazoline-6-sulfonic acid) (ABTS) scavenging potential of soybean as compared to unfermented soybean (Figure 2). The results revealed the maximum scavenging was found in soybean fermented with 121.18% increase in soybean fermented with 1% *B. amyloliquefaciens* RWL-1 followed by 71.30% and 35.67% increase in soybean fermented with 3% and 5% respectively, as compared to unfermented soybean (Figure 2).

### 2.3. Total Amino Acids Composition

In our study, *B. amyloliquefaciens* RWL-1 fermentation significantly improved amino acids composition of fermented soybean as compared to non-fermented soybean. The increased amount of total amino acids were recorded in soybean fermented with 1% *B. amyloliquefaciens* RWL-1 with 329.26% increase, followed by 3% and 5% *B. amyloliquefaciens* RWL-1 with 185.35% and 57.86% increase respectively, while the less amount was recorded in unfermented soybean (Table 1). More specifically, aspartic acid, threonine, glutamic acid, glycine, alanine, cysteine, valine, methionine, isoleucine, leucine, tyrosine, phenylalanine, lysine, histidine, arginine, and proline was increased in soybean fermented with 1% *B. amyloliquefaciens* RWL-1 with 222.22%, 160.56%, 290.43%, 250.89%, 199.73%, 58.52%, 183.29%, 511.11%, 221.20%, 314.23%, 123.17%, 226.58%, 216.21%, 402.30%, 105.39%, and 255.13% followed by soybean fermented with 3% *B. amyloliquefaciens* RWL-1 with 211.11%, 53.89%, 141.74%, 114.29%, 145.7%, 215.66%, 155.06%, 324.07%, 142.41%, 178.47%, 112.60%, 126.88%, 81.08%, 399.23%, 62.63%, and 166.57% and soybean fermented with 5% *B. amyloliquefaciens* RWL-1 with 68.89%, 13.89%, 54.35%, 66.07%, 36.29%, 9.89%, 30.35%, 85.19%, 55.59%, 59.85%, 29.67%, 25.43%, 34.75%, 13.08%, 44.06%, and 45.75%, respectively, as compared with non-fermented soybean (Table 1). Only serine was reduced with 13.79%, 1.72%, and 1.72% decreased in soybean fermented with 1%, 2%, and 5% *B. amyloliquefaciens* RWL-1 respectively as compared with non-fermented soybean. (Table 1)

### 2.4. Determination of Isoflavones

In the current study, isoflavones contents were estimated in fermented soybean in comparison with non-fermented soybean. In our study, the soybean fermented with *B. amyloliquefaciens* RWL-1 displayed enhanced isoflavones contents as compared to non-fermented soybean (Table 2). The results of the current study revealed that fermentation of soybean with various percentages of *B. amyloliquefaciens* RWL-1 significantly increased the total isoflavones. Total isoflavones were significantly improved with 32.71% increase in soybean fermented with 1% *B. amyloliquefaciens* RWL-1 followed by 18.47% increase in soybean fermented with 3% *B. amyloliquefaciens* RWL-1, while the soybean fermented with 5% *B. amyloliquefaciens* RWL-1 displayed 4.49% decrease in total isoflavones contents as compared to non-fermented soybean (Table 2). More specifically, significantly increased amount of daidzin, genistin, and glycitin with 96.13%, 53.21%, and 225.40% increase were recorded in soybean fermented with 1% *B. amyloliquefaciens* RWL-1 with, followed by 84.96%, 36.94%, and 189.68% increase respectively, in soybean fermented with 3% *B. amyloliquefaciens* RWL-1 and with 64.60%, 11.54%, and 91.95% increase in soybean fermented with 5% *B. amyloliquefaciens* RWL-1, respectively, as compared to non-fermented soybean (Table 2). While, daidzein, glycitein and genistein were significantly reduced with 61.18%, 72.98%, and 64.15% decrease, respectively, upon fermentation with 5% *B. amyloliquefaciens* RWL-1 followed by 51.53%, 48.34%, and 59.13% decrease in soybean fermented with 3% *B. amyloliquefaciens* RWL-1 43.13%, 39.78%, and 36.95% decrease in soybean fermented with 1% *B. amyloliquefaciens* RWL-1, respectively, as compared to non-fermented soybean (Table 2).

### 2.5. Assessment of Minerals

In the current study, 11 minerals viz. calcium (Ca), copper (Cu), iron (Fe), potassium (K), magnesium (mg), manganese (Mn), sodium (Na), nickel (Ni), lead (Pb), arsenic (As), and zinc (Zn) were estimated in fermented and non-fermented soybean. The fermentation with various percentages of *B. amyloliquefaciens* RWL-1 revealed improved mineral contents of soybean as compared to non-fermented soybean (Table 3). The highest total mineral contents were observed in soybean fermented with 5% *B. amyloliquefaciens* RWL-1 with 28.68% increase followed by 12.23% and 5.76% increase in soybean fermented with 3% and 1%, respectively as compared to non-fermented soybean. More specifically the soybean fermented with 5% *B. amyloliquefaciens* RWL-1 enhanced the level of Ca, Fe, K, Mg, Mn, Na, and Zn by 10.14%, 181.07%, 28.83%, 38.70%, 100.38%, 315.86%, and 24.62% increase followed by soybean fermented with 3% *B. amyloliquefaciens* RWL-1 resulting enhanced Ca (7.90%), Fe (89.37%), K (13.74%), Mg (15.82%), Mn (98.16%), Na (59.34%), and Zn (15.89%), subsequently, soybean fermented with 1% *B. amyloliquefaciens* RWL-1 resulting enhanced Ca (4.57%), Fe (40.01%), K (3.78%), Mg (12.03%), Mn (95.87%), Na (127.311%), and Zn (15.89%) as compared to non-fermented soybean (Table 3). However, the Ni and Cu were significantly decreased in soybean upon fermentation. The reduced amount of Cu with 34.99% decrease was recorded in soybean fermented with 1% *B. amyloliquefaciens* RWL-1 followed by 14.58% and 13.53% decrease in soybean fermented with 3% and 5% *B. amyloliquefaciens* RWL-1 respectively as compared to non-fermented soybean (Table 3). Likewise, reduced amount of Ni with 68.766% decrease was recorded in soybean fermented with 1% *B. amyloliquefaciens* RWL-1 followed by 35.98% and 15.68% decrease in soybean fermented with 3% and 5% *B. amyloliquefaciens* RWL-1 respectively as compared to non-fermented soybean (Table 3). Moreover, As and Pb were not detected in fermented and non-fermented soybean.

## 3. Discussion

Fermented food has been a rich source of nutrients for a long time. Fermented foods are considered a rich source of various health beneficial constituents such as minerals, vitamins, antioxidants, folic acids, and fibers [21]. The consumption of fermented foods boosts our immune system, fights against inflammation, assists digestion, strengthens bones, enhances energy, balance cholesterol level, maintains the proper blood pressure and contributes in maintaining the youthful skin [22]. The fermented foods are generally available in the food market as dairy products that contain live microbe and also available in drug store as tablets and supplements which contain lyophilized microbes or its constituents which promote health benefits [23].

Soybean is considered a rich source of proteins, fatty acids, minerals, vitamins, isoflavones, amino acids, and carbohydrates [24]. Soybeans are consumed either directly as unfermented or indirectly as fermented soybean [25]. Various types of fermented soybeans are consumed and are very famous in Asian countries. Among other fermented soybeans, CGJ are popular fermented soybean consumed in South Korea and is considered a rich source of health beneficial constituents [12,13]. Keeping in view the nutritional and health beneficial potential of fermented soybean (Cheonggukjang (CGJ)), the current study was undertaken using *B. amyloliquefaciens* RWL-1. Seed-borne endophytic *B. amyloliquefaciens* RWL-1 was isolated from rice seed and has been reported for antifungal activity and various secondary metabolites and novel compound production [17,18,19,20]. The soybean fermented with various concentrations of *B. amyloliquefaciens* RWL-1 enhanced the total phenolic contents as compared with non-fermented soybean (Figure 1). Total phenolic content is a group of important bioactive metabolites, often investigated in scientific studies. Phenolic contents are considered important in functional food and pharmaceutics industries because of their free radical scavenging potential [26]. Similar results of enhanced total phenolic contents in soybean fermented with various probiotic *B. subtilis* has been previously reported [12,13]. Moreover, similar results of enhanced TPC of fermented soybean were 644 ± 11.20 lg/g after 48 h fermentation is reported [27]. Likewise, another study reported 21% and 35% increase in TPC in soybean fermented with naturally occurring bacteria (soybean was left to ferment by naturally occurring microbes) and *B. subtilis* TN51, respectively [28]. However, the results of the current study reported comparatively increased (181.50%) amount of TPC in soybean fermented with 1% of *B. amyloliquefaciens*. The differences might be due to difference in soybean cultivars and microbes concentrations used as a starter [12,13,29].

2,2-diphenyl-1-picrylhydrazyl (DPPH) and 2,2-azinobis (3-ethylbenzothiazoline-6-sulfonic acid) (ABTS) are the commonly used to assess antioxidant activities [30]. DPPH is a cationic free radical and is based on an electron based reaction which is considered as an important arbitrator for investigating the antioxidant potentials of any compound. The DPPH scavenging potential is indicated by the degree of reduction and resulted in discoloration of tested samples [31]. The ABTS is generated by reacting with a strong oxidizing agent (e.g., potassium permanganate or potassium persulfate) with the ABTS salt [30]. The reduction of the blue-green ABTS radical by hydrogen-donating antioxidants is measured by the suppression of its characteristic long-wave absorption spectrum. However, the ABTS assay measures the relative ability of antioxidants to scavenge the ABTS generated in aqueous phase [30]. In the current study, the fermentation with *B. amyloliquefaciens* RWL-1 improves the antioxidants potential of soybean used in CGJ (Figure 2). Similar results of improved antioxidants potential of fermented soybean have been reported by Ali et al. [13]. They reported the improvement in DDPH radical scavenging activity by 94.24% ± 0.83% increase of soybean fermented with 1% *B. subtilis*. Similarly, Cho et al. [32] reported the increased DPPH radical scavenging activity by 53.6%–93.9% after 60 h in soybean fermented with *B. subtilis* CS90. Likewise, Shon et al. [33] also reported DPPH radical scavenging activity by 69%–87% of soybean fermented with *B. megaterium*. However, comparatively, soybean fermentation with 1% *B. amyloliquefaciens* RWL-1 in current study revealed higher DPPH radical scavenging activity with 323.39% increase. The antioxidants data of the current study suggest that soybean fermented with 1% *B. amyloliquefaciens* exhibit enhanced and improved antioxidants. Similarly, ABTS assessment of soybean fermented with *B. amyloliquefaciens* exhibited increased ABTS scavenging activity as shown in Figure 2. A similar result of enhanced ABTS scavenging activity of soybean fermented with conventional probiotic *B. subtilis* by 86% is reported [13]. Likewise, Shin et al. [34] also reported the enhanced ABTS scavenging potential of soybean fermented with *B. subtilis*. Another study reported 91.06% and 81.12% ABTS scavenging activities in soybean fermented with *Seoritae* and *Seormoktae* sp. [35]. The current study revealed the maximum scavenging with 121.18% increase in soybean fermented with 1% *B. amyloliquefaciens* RWL-1. Recently, many researchers have linked the increased antioxidants potential of fermented soybean with flavonoids contents of fermented soybean [36]. The flavonoids contents of fermented soybean which could respond to generates ions which further extinguish free radicals and resulting in the axing of free radicals chain reaction [37].

The term amino acid refers to the structural unit of proteins and natural peptides. They are also precursors of very important non-proteinogenic biological compounds [38]. The increase in amino acids contents of soybean fermented with *B. amyloliquefaciens* RWL-1 is in line with the results is reported [13]. They reported the enhanced amino acids levels in soybean fermented with probiotic *B. subtilis* (KCTC 13241). They reported the highest amount of total amino acids (7.430 ± 0.62 mg/g) in soybean fermented 1% *B. subtilis* among the various concentration of conventional probiotic *B. subtilis*, however in the current we found the highest amount (204.77 ± 6.38 mg/0.1g) of total amino acids with comparatively higher percentage of increase in soybean fermented with 1% *B. amyloliquefaciens* RWL-1. (Table 1). Similarly another study reported by Sarkar et al. [39], revealed a 60-fold increase in amino acid of fermented soybean with *B. subtilis*. Likewise, Dajanta et al. [40] investigated the essential amino acids and free amino acids in traditionally fermented soybean (thua nao) and revealed the essential amino acid profile of thua nao within the range of 20.23% to 35.41% with most abundantly Trp, Leu, and Lys contents. Similarly in some other studies focused on the amino acids profile of chungkukjang prepared with *Bacillus* sp. revealed the presence of amino acids at considerable amount of 53% [41]. The increase in amino acid contents in soybean fermented with *B. amyloliquefaciens* RWL-1 is linked with amino acids production potential of *B. amyloliquefaciens* RWL-1 [42]. The amino acids presented as essential nutrients for health-promoting activities [43]. Therefore, their supplementation or enhancement in food is greatly required. The amino acids data of the current study suggest that soybean fermented with 1% *B. amyloliquefaciens* are a rich source of protein and exhibit favorable amino acids profile.

Isoflavones contents of soybean have been widely known for their beneficial health properties and have been recognized as important dietary supplements [44]. Soybean contains β-D-glucosides (daidzin, glycitin, and genistin) and aglycones (daidzein, glycitein, and genistein) isoflavones. In the current study, β-D-glucosides (daidzin, glycitin, and genistin) were significantly increased upon fermentation with *B. amyloliquefaciens* RWL-1 while the aglycones (daidzein, glycitein, and genistein) were significantly decreased upon fermentation with *B. amyloliquefaciens* RWL-1 (Table 2). Generally, soybeans are composed of glycone and aglycone as two classes of isoflavones. However, the compositions of various types of glycine and aglycone isoflavones vary in soybean food because of their varieties and processing techniques [45]. Generally, isoflavones are present in glycone form and transformed into aglycone during microbial fermentation [46]. The transformation of various isoflavones to other compounds as a result of high temperature and high steam pressure during fermentation is reported [47]. The loss and reduction in isoflavones are possibly due to thermal degradation and leaching in water [33]. The increased total isoflavones contents of fermented soybean are widely reported [12,48]. However, the changes in the Isoflavones contents of fermented soybean might be due to the differences in processing techniques and temperature [34]. The similar results of enhanced daidzin, glycitin, and genistin while reduced daidzein, glycitein, and genistein upon fermentation is reported by Ali et al. [13] Likewise, Cho et al. [32] reported 64% the decrease in isoflavones in fermented soybean. Hwang et al. [35] reported the increase in aglycone isoflavones while the decrease in glycone isoflavones in fermented soybean. While another study reported that aglycone form of isoflavones could not be expected during tempeh (Indonesian fermented soybean) fermentation [49]. Conversely, another study reported increased aglycone isoflavones only at the beginning of fermentation but did not increase further [50]. Likewise, Yang et al. [51] reported that agylcone contents in fermented soybean did not increase upon inoculation with *B. subtilis*. These contrasting results suggest that changes in isoflavone concentrations and profiles in fermented soybean differ due to thermal processing, temperature, fermentation period, and microorganisms [52].

Fermentation represents a technological alternative to improve cereals functional and nutritional properties [53]. In the current study, the macro and micro nutrients were significantly enhanced in soybean fermented with *B. amyloliquefaciens* RWL-1 (Table 3). The enhanced minerals contents in fermented soybean are might be due to the bacterial involvement and bacterial interaction with metabolites breakdown [13,54]. The similar results of increased nutritional value by degrading and decreasing anti-nutritional and improving the bioavailability of health beneficial minerals in soybean upon fermentation is reported [13]. However, the total mineral contents in soybean fermented with conventional *B. subtilis* were 13,816 ± 23 mg/kg while the total mineral contents in fermented soybean was significantly improved and revealed 16,363.42 ± 17 mg/kg with 32.71% increase.

## 4. Materials and Methods

### 4.1. Chemicals

All chemicals, reagents, solvents, and standards used in the experiments were obtained from Sigma Chemical Co. (St. Louis, MO, USA). Doubled distilled deionized water was used in all experiments. All chemicals were of analytical grade.

### 4.2. Microorganism and Soybean Material

Seed-borne endophytic *B. amyloliquefaciens* RWL-1 used in current experiment was previously isolated from rice seeds and its sequence is submitted to NCBI (Bethesda, MD, USA) under the accession number KR677384 and reported for antifungal potential and various secondary metabolites production [17,18,19,20]. In the current experiment *B. amyloliquefaciens* RWL-1 was grown in LB medium (Luria–Bertani) at 28 °C.

Soybean cultivar (Aga 3) was selected based on the results of our previously reported work [12] and was obtained from the genetic resource center, Kyungpook National University, Daegu, South Korea.

### 4.3. Preparation of Fermented Soybeans

Preparation of fermented soybeans (FS) was carried out by the method of Ali et al. [13] with some modifications. Soybeans were sorted, washed, and soaked in distilled water for 12 h at room temperature. After draining, soybeans were heated for 15 min at 121 °C. The steamed soybeans were left to stand for 2 h at 40 ± 1 °C to cool down. Then cooked soybeans were inoculated with 1%, 3%, and 5% (*v*/*w*) *B. amyloliquefaciens* RWL-1 and fermented in an incubator for 60 h at 40 ± 2 °C. This experiment was repeated three times.

### 4.4. Preparation of Sample Extraction

Extractions were carried out by the method of Ali et al. [12,13] with some modifications. Briefly, 10 g of each ground freeze-dried sample was shaken in 50 mL of 80% methanol at 37 °C for 24 h. The incubated extract centrifuged at 3000 rpm for 20 min. The supernatant was then filtered through a 0.45-μm Millipore PVDF filter (Schleicher and Schuell, GmbH, Dassel, Germany). The filtrate was used for assays of polyphenolic compounds and antioxidant activities.

### 4.5. Determination of Total Phenolic Contents

The Folin–Ciocalteu method was used to determine the amount of total phenolic contents according to the method described by Ali et al. [12] Fifty microliters of methanolic extract was added to 1 mL aqueous solution of 2% sodium carbonate (Na_2_Co_3_) and was left for 3 min. The mixture was mixed with 50 µL of 1 N Folin–Ciocalteu reagent and incubated in the dark for 30 min at room temperature. The absorbance of the reaction mixture was read at 750 nm using a Multiskan GO Microplate Spectrophotometer (Thermal Fischer Scientific, Vantaa, Finland). The standard calibration curve was plotted using gallic acid. The total phenolic contents were expressed as milligram gallic acid equivalent per gram of dried sample (mg GAE/g).

### 4.6. DPPH Radical Scavenging Activity

The DPPH radical-scavenging effect of non-fermented and fermented soybean was measured as described by Bilal et al. [55] with some modifications. Briefly, freshly prepared DPPH solution in methanol was used for the experiment. A mixture of equal volumes of methanol extracted samples and freshly prepared 0.1% DPPH solution was left in dark for 30 min at ambient temperature. After the incubation period, the absorbance was measured at 517 nm. The inhibitory percentage of DPPH was calculated according to the following equation:

Scavenging effect (%) = 1 (absorbance of sample/absorbance of control) × 100

### 4.7. ABTS Radical-Scavenging Activity

The antioxidant activity of the fermented and non-fermented soybean was measured by the ABTS radical cation decolorization assay involving preformed ABTS radical cations [55]. ABTS was dissolved in 0.01 M sodium phosphate buffer to a 7 mM concentration. The ABTS was produced by reacting ABTS stock solution with 2.45 mM potassium persulfate (final concentration) and allowing the mixture to stand in the dark at room temperature for 12 to 16 h before use. The stock solutions of the soybean extracts in methanol were diluted so that a 2 μL aliquot of each dilution was used in the assay. After the addition of 200 μL of the diluted ABTS solution to the antioxidant compounds or Trolox standards in methanol, samples were taken at 37 °C exactly 7 min after initial mixing. The absorbance of the resulting solution was measured at 734 nm.

### 4.8. Free Amino Acid Analysis

The free amino acid composition of freeze-dried samples was determined according to our previous study [18]. Briefly, the amino acid composition in all treatments was determined with a Hitachi Amino Acid Analyzer (L-8900, Hitachi, Tokyo, Japan) after hydrolysis of 100 mg protein with 6 M HCl at 110 °C for 24 h. An amino acid standard mixture solution for automatic amino acid analysis (Type H, Wako Pure Chemical Industries Ltd., Osaka, Japan) was used for the quantification of endogenous amino acid content. All of the samples were run in triplicate and expressed in mg/0.1 g dry weight.

### 4.9. Determination of Isoflavones

Isoflavone determination was carried out according to the protocol described by Ali et al. [12] Briefly, 1 g of grinded sample was defatted and extracted with 5 mL Hexane and extraction buffer (5 mL acetonitrile, 4.5 mL water, and 0.5 mL of the internal standard THB (0.5 mg/mL), respectively. The extracted samples were centrifuged at 10,000× *g* to collect the supernatant. The obtained supernatant was concentrated by evaporation, filtered and injected to HPLC equipped with a Symmetry C18 column (Water, Milford, MA, USA). The UV detector was stabilized with a mobile phase (A: Acetonitrile, B: HPLC water (1% acetic acid), A: 5% (1 min), 5%→35% (50 min), 35%→5% (5 min), 5% (15 min) at a flow rate of 1.0 mL/min). The effluent was detected at 254 nm. The isoflavones were identified by their retention times of standard and were calculated by comparing their peak areas with those of standards.

### 4.10. Assessment of Minerals

Mineral content was determined following the method of Ali et al. [13] with some modifications. Sample powder (0.5 g) and HNO_3_ (15.0 mL) were mixed into a cup. The mixture was diluted with an equal volume of distilled water. Mineral concentrations were determined using an inductively coupled plasma atomic emission spectrometer (ICP AES: Varian Vista, Varian Australia, Victoria, Australia).

### 4.11. Statistical Analysis

The data collected from the triplicate experiments were pooled together and expressed as mean ± SD. The collected data from the different treatments have been equally compared and subjected to Duncan’s multiple range tests (DMRT) using SAS (Version 9.3, SAS Institute Inc., Cary, NC, USA) to reveal statistical difference among treatments. Moreover, GraphPad Prism (Version 6.0, San Diego, CA, USA) was used for graphical presentations.

## 5. Conclusions

Fermentation is considered an alternative to improve the nutritional properties of functional food. The current study showed the potential of *B. amyloliquefaciens* RWL-1 at 1% concentration in nutrient enrichment, enhanced antioxidants properties, amino acids enhancement and improvement of isoflavones contents in fermented soybean. These properties provide attractive possibilities for further utilization of this strain in order to improve the health promoting benefits of functional food. Moreover, further studies are needed for the characterization of specific genes and bioactive compounds responsible for these beneficial properties.

## Figures and Tables

**Table 1:** Amino acids contents in non-fermented and fermented soybean with various concentrations (1%, 3%, and 5%) of *B. amyloliquefaciens* RWL-1.

Amino Acid mg/0.1 g	Control	1% RWL-1	3% RWL-1	5% RWL-1
aspartic acid	0.45 ± 0.04 ^c^	1.54 ± 0.10 ^a^	1.40 ± 0.14 ^a^	0.76 ± 0.03 ^b^
Threonine	1.8 ± 0.01 ^d^	4.69 ± 0.13 ^a^	2.77 ± 0.09 ^b^	2.05 ± 0.31 ^c^
Serine	0.58 ± 0.03 ^a^	0.50 ± 0.06 ^b^	0.57 ± 0.01 ^a^	0.57 ± 0.01 ^a^
glutamic acid	2.3 ± 0.01 ^d^	8.98 ± 0.62 ^a^	5.56 ± 0.30 ^b^	3.55 ± 0.04 ^c^
Glycine	1.12 ± 0.02 ^d^	3.93 ± 0.15 ^a^	2.4 ± 0.23 ^b^	1.86 ± 0.43 ^c^
Alanine	7.44 ± 0.33 ^d^	22.30 ± 0.70 ^a^	18.28 ± 0.46 ^b^	10.14 ± 0.50 ^c^
Cysteine	3.64 ± 0.37 ^c^	5.77 ± 0.28 ^b^	11.49 ± 0.32 ^a^	4.00 ± 0.13 ^c^
Valine	4.25 ± 0.21 ^d^	12.04 ± 0.19 ^a^	10.84 ± 0.15 ^b^	5.54 ± 0.36 ^c^
Methionine	1.08 ± 0.09 ^d^	6.60 ± 0.24 ^a^	4.58 ± 0.39 ^b^	2.00 ± 0.18 ^c^
Isoleucine	3.49 ± 0.23 ^d^	11.21 ± 0.49 ^a^	8.46 ± 0.23 ^b^	5.43 ± 0.32 ^c^
Leucine	5.48 ± 0.37 ^d^	22.70 ± 0.38 ^a^	15.26 ± 0.37 ^b^	8.76 ± 0.44 ^c^
Tyrosine	2.46 ± 0.25 ^c^	5.49 ± 0.31 ^a^	5.23 ± 0.29 ^a^	3.19 ± 0.35 ^b^
Phenylalanine	3.46 ± 0.25 ^d^	11.30 ± 0.59 ^a^	7.85 ± 0.11 ^b^	4.34 ± 0.38 ^c^
Lysine	2.59 ± 0.31 ^d^	8.19 ± 0.26 ^a^	4.69 ± 0.23 ^b^	3.49 ± 0.30 ^c^
Histidine	1.30 ± 0.08 ^b^	6.53 ± 0.39 ^a^	6.49 ± 0.46 ^a^	1.47 ± 0.07 ^b^
Arginine	4.63 ± 0.26 ^d^	9.51 ± 0.34 ^a^	7.53 ± 0.37 ^b^	6.67 ± 0.37 ^c^
Proline	3.41 ± 0.25 ^d^	12.11 ± 0.52 ^a^	9.09 ± 0.64 ^b^	4.97 ± 0.38 ^c^
Total Amino Acids	47.70 ± 3.25 ^d^	204.77 ± 6.38 ^a^	136.11 ± 4.97 ^b^	75.30 ± 3.49 ^c^

Values in rows followed by different letters (a, b, c, and d) are significantly different at *P* < 0.05 revealed by DMRT tests.

**Table 2:** Isoflavones quantification in non-fermented and fermented soybean with various concentrations (1%, 3%, and 5%) of *B. amyloliquefaciens* RWL-1.

Isoflavone mg/kg	Control	1% RWL-1	3% RWL-1	5% RWL-1
Daidzin	154.32 ± 1.76 ^d^	302.67 ± 3.12 ^a^	285.44 ± 2.54 ^b^	254.02 ± 2.74 ^c^
Genistin	260.56 ± 3.14 ^d^	399.21 ± 2.43 ^a^	356.82 ± 3.92 ^b^	290.64 ± 4.51 ^c^
Glycitin	25.98 ± 2.45 ^d^	84.54 ± 1.32 ^a^	75.26 ± 1.33 ^b^	49.87 ± 0.48 ^c^
Daidzein	183.72 ± 3.37 ^a^	104.48 ± 4.83 ^b^	89.04 ± 1.65 ^c^	71.32 ± 1.12 ^d^
Glycitein	126.86 ± 2.28 ^a^	76.39 ± 2.35 ^b^	63.53 ± 2.74 ^c^	34.27 ± 0.98 ^d^
Genistein	42.65 ± 2.05 ^a^	26.89 ± 0.66 ^b^	17.43 ± 1.78 ^c^	15.29 ± 1.05 ^d^
Total isoflavones	749.09 ± 4.72 ^c^	994.18 ± 6.91 ^a^	887.52 ± 6.35 ^b^	715.41 ± 4.69 ^d^

Values in rows followed by different letters (a, b, c, and d) are significantly different at *P* < 0.05 revealed by DMRT tests.

**Table 3:** Minerals analysis in non-fermented and fermented soybean with various concentrations (1%, 3%, and 5%) of *B. amyloliquefaciens* RWL-1.

Minerals (mg/kg)	Control	1% RWL-1	3% RWL-1	5% RWL-1
Calcium	1754.41 ± 12 ^d^	1834.64 ± 19 ^c^	1893.32 ± 37 ^b^	1932.35 ± 25 ^a^
Copper	23.72 ± 0.15 ^a^	15.42 ± 0.93 ^c^	20.26 ± 0.14 ^b^	20.51 ± 1.13 ^b^
Iron	44.23 ± 1.54 ^d^	61.93 ± 0.73 ^c^	83.76 ± 1.91 ^b^	124.32 ± 1.24 ^a^
Potassium	9945 ± 34.03 ^d^	10321 ± 23.32 ^c^	11322 ± 24.32 ^b^	12813 ± 43.28 ^a^
Magnesium	787.54 ± 23.03 ^d^	882.32 ± 11.36 ^c^	912.16 ± 31.2 ^b^	1092.33 ± 32.5 ^a^
Manganese	47.29 ± 0.14 ^c^	92.63 ± 2.76 ^b^	93.71 ± 4.76 ^a,b^	94.76 ± 3.43 ^a^
Sodium	58.51 ± 3.62 ^d^	133.87 ± 3.54 ^b^	93.23 ± 2.32 ^c^	243.32 ± 3.54 ^a^
Nickel	7.78 ± 1.24 ^a^	2.43 ± 0.07 ^d^	4.98 ± 1.12 ^c^	6.56 ± 1.32 ^b^
Zinc	29.32 ± 1.76 ^c^	33.98 ± 1.79 ^b^	33.98 ± 1.79 ^b^	36.54 ± 1.03 ^a^
Total Minerals	12715.50 ± 41 ^d^	13449.18 ± 27 ^c^	14271.04 ± 36 ^b^	16363.42 ± 17 ^a^

Values in rows followed by different letters are significantly different at *P* < 0.05 revealed by DMRT tests.
